# Efficacy and safety of artemether + lumefantrine, artesunate + sulphamethoxypyrazine-pyrimethamine and artesunate + amodiaquine and sulphadoxine-pyrimethamine + amodiaquine in the treatment of uncomplicated *falciparum* malaria in Bangui, Central African Republic: a randomized trial

**DOI:** 10.1186/1475-2875-13-9

**Published:** 2014-01-07

**Authors:** Djibrine Djallé, Siméon P Njuimo, Alexandre Manirakiza, Rémi Laganier, Alain Le Faou, Christophe Rogier

**Affiliations:** 1 Institut Pasteur de Bangui, BP 923 Bangui, Central African Republic; 2 Institut de Recherche Biomédicale des Armées, Antenne de Marseille, Marseille, France; 3 Laboratoire de Virologie, Centre Hospitalier Universitaire de Nancy, Hôpital de Brabois Adultes, 54511 Vandoeuvre-lès-Nancy, Cedex, France; 4 Institut Pasteur de Madagascar, BP 1274, 101 Antananarivo, Madagascar

**Keywords:** ACT, Efficacy, Safety, Bangui, Randomized trial

## Abstract

**Background:**

The efficacy of artemisinin-based combination therapy (ACT) has been established. The objective of the present study was to compare the efficacy and safety in the Central African Republic (CAR) of three commercially available artemisinin-based combinations, artemether + lumefantrine (AL), artesunate + sulphamethoxypyrazine–pyrimethamine (AS-SMP) and artesunate + amodiaquine (AS-AQ), with those of sulphadoxine–pyrimethamine + amodiaquine (SP-AQ), which was the first-line reference treatment in the country from 2004, until it was replaced by ACT in 2006 in accordance with changes in international recommendations based on resistance identified in other regions.

**Methods:**

Children aged six to 59 months with uncomplicated *Plasmodium falciparum* malaria were recruited in Bangui, the capital of the CAR. The 251 patients selected were randomly assigned to receive AL (*n* = 60), AS-SMP (*n* = 58), AS-AQ (*n* = 68) or SP-AQ (*n* = 65) and were followed up for 28 days. Clinical outcome was classified according to the standard 2003 World Health Organization protocol.

**Results:**

At day 28, the cure rates in a per-protocol analysis were 92% (48/52) with AL, 93% (50/54) with AS-SMP, 93% (55/59) with AS-AQ and 100% (57/57) with SP-AQ, with no statistically significant difference between the four treatments. Defervescence was significantly faster with AS-AQ than with AL (*p* <0.035). Fatigue was reported significantly more frequently by patients receiving AQ than by those treated with AS-SMP or AL (*p* = 0.006). All the other adverse events reported were mild, and no significant difference was noted by treatment.

**Conclusion:**

The three artemisinin-bsed combinations show similar, satisfactory results, comparable to that with SP-AQ. This evaluation is the first conducted in CAR since the official introduction of ACT. It should guide the National Malaria Control Programme in choosing the appropriate ACT for treatment of uncomplicated *P. falciparum* malaria in the future.

## Background

Malaria remains an important killer, particularly among children in sub-Saharan Africa [1]; it also slows the development of children and national economies [2]. Effective drugs and adequate treatment compliance therefore play a key role in lowering the malaria burden. As recommended by the World Health Organization (WHO) [3], artemisinin-based combination therapy (ACT) has become the reference treatment for uncomplicated malaria in most endemic areas [4-6]. Although resistance to artemisinin was recently observed in western Cambodia [7,8], ACT has been found to be effective all over the world, generally with good clinical and parasitological responses [1,9-12]. Nevertheless, side effects, ease of administration, cost and duration of treatment are important considerations in selecting anti-malarial treatment.

In the Central African Republic (CAR), a high prevalence of chloroquine-resistant *Plasmodium falciparum* necessitated a switch to sulphadoxine–pyrimethamine + amodiaquine (SP-AQ) as the recommended first-line treatment for uncomplicated *P. falciparum* malaria in 2004 [13,14]. In line with WHO recommendations, SP-AQ was replaced by ACT in 2006 [15]. Unfortunately, ACT was still not available in a number of health facilities in 2006 [16], and chloroquine and SP remained in common use at least until 2009 (personal observation), despite a high prevalence of resistance to these drugs in *P. falciparum*[17]. Medical doctors and nurses lacked confidence in the efficacy of ACT, as some patients returned with positive thick smears more than 72 hours after treatment with artemether + lumefantrine (AL); therefore, they continued to prescribe anti-malarial drugs that were not recommended (personal observation). These treatment failures may have been due to lack of communication or non-respect of the recommendation that this drug be taken during a fatty meal to ensure good bioavailability of artemether [18,19].

Only two clinical evaluations of anti-malarials have been conducted in the CAR, besides efficacy studies of chloroquine, AQ and various combinations with SP [13] and an evaluation of artemether as mono-treatment for *P. falciparum* malaria*,* which is no longer recommended [20]. Thus, the switch to ACT in 2006 was based only on the WHO recommendation and on clinical studies in other African countries.

The objective of the present study was to assess the efficacy and the safety of three artemisinin-based combinations AL, artesunate (AS) + sulphamethoxypyrazine-pyrimethamine (SMP) (AS + SMP is not a combination recommended by WHO but is available on Central African markets and is widely used) and AS + AQ in the CAR and to compare their efficacy with that of the SP + AQ combination that was previously used as first-line treatment in the country.

## Methods

The study was carried out between May 2008 and May 2009 in two selected urban health centres, the Paediatric Complex and the Saint Joseph, Health Centre, in Bangui, the capital of the CAR, which draw children from all districts of the city. Bangui is located on the Oubangui River in the south of CAR, north of the Democratic Republic of the Congo (geographical coordinates, 4.29 N, 18.35 E). The climate is tropical, and the rainy season lasts from April to November. The temperature varies from 19 to 32°C, with an average of 26°C. Malaria transmission occurs throughout the year, with peaks at the beginning and end of the rainy season. Malaria is hyperendemic, and the predominant parasite species is *P. falciparum.*

### Study design

The study was a prospective randomized open-label trial of the *in vivo* efficacy and safety of AL, AS-SMP and AS-AQ in comparison with SP-AQ. Patients were followed up for 28 days after treatment, with a clinical examination and laboratory analysis. The study was carried out according to the recommendations of WHO for surveillance of the efficacy of anti-malarial drugs [21].

### Patients

Between May 2008 and May 2009, febrile patients aged six to 59 months presenting to the two health facilities were enrolled in the study if informed consent was provided by a parent or guardian and if they had an axillary temperature ≥ 37.5°C or a history of fever in the preceding 24 hours, a *P. falciparum* monoinfection, parasitaemia of 2,000–200,000 asexual parasites per μl of blood, no known intolerance (e g, allergy) to the study drugs, no use of any component of the study drugs within the 28 days before enrolment, no cause of fever other than malaria and no danger signs (unable to sit or stand, unable to drink or breastfeed, lethargic or unconscious, recent history of convulsions, persistent vomiting) related or not to severe and complicated *falciparum* malaria, according to the WHO definition [22]. To be included, participants had to be able to come to the health facility for follow-up. Parents or guardians were asked by a physician about symptoms, duration of illness, previous anti-malarial therapy and other medications. Children were examined for pallor, jaundice and any other danger sign, and their axillary temperature and weight were measured.

Randomized codes, computer-generated by a third party not involved in the study, were attributed to patients in numerical sequence by the investigators, permitting their randomized allocation to one of the four treatment groups.

### Treatments

All drugs were from manufacturers that comply with good manufacturing practice. AL (Coartem®) was provided by Novartis, Switzerland, AS-SMP (Coarinate®) by DafraPharma, Belgium, AS-AQ (Coarsucam®) by Sanofi Aventis, France, and SP (Fansidar®)–AQ by IDA, Netherlands.

Group 1 received AL, which is a combination of 20 mg artemether and 120 mg lumefantrine per tablet. AL was administered according to body weight (5–14 kg, one tablet; 15–24 kg, two tablets; 25–34 kg, three tablets; ≥ 35 kg, four tablets) in six consecutive doses: the first dose at diagnosis (day 0), the second eight hours later (day 0) and then four doses at 12-hour intervals over the following two days (days 1 and 2). The second dose on day 0 was administered at home by study staff.

Group 2 received AS-SMP, which is available as “junior” and infant treatment packs consisting of three tablets of AS and three of SMP, each of which is given at the same time. The junior tablets contain 100 mg AS and 250 mg SMP with 12.5 mg of pyrimethamine, and the infant tablets contain 50 mg AS and 125 mg SMP with 6.25 mg pyrimethamine. The packs were prescribed according to weight: children weighing 20–40 kg were given junior tablets, those weighing 13–20 kg received one and a half infant tablets, and those weighing 5–8 kg received one infant tablet. The treatment consisted of three doses at 24-hour intervals (0 hours, 24 hours, 48 hours).

Group 3 received AS-AQ, which is presented in three dosages per tablet: for large children (weighing 18–36 kg), 100 mg AS and 270 mg AQ; for small children (weighing 9–18 kg), 50 mg AS and 135 mg AQ; and for infants (weighing 4.5-9 kg), 25 mg AS and 67.5 mg AQ. Treatment consists of three tablets, taken one at a time at 24-hour intervals (0 hours, 24 hours, 48 hours).

Group 4 received SP-AQ, which exists in only one version, with two tablets, one containing 500 mg sulphadoxine and 25 mg pyrimethamine and one containing 200 mg AQ hydrochloride. The dosage was determined by weight: children weighing ≤10 kg received half a tablet, those weighing 10–20 kg received one tablet, those weighing 21–30 kg were given one and a half tablets, and those weighing 31–40 kg received two tablets in one dose. The dose of AQ was 8 mg/kg per day over three days given as single doses at 24-hour intervals.

Unless otherwise indicated, all drug doses were administered at the health centre by the study team, with the exception of the second dose of AL, which was administered in the patient’s home by the study team. For all groups, a full dose was re-administered if the patient either spat out the drug or vomited it within 30 min; half the dose was re-administered if the patient vomited between 30 min and one hour after administration. If the patient vomited again, he or she was given another anti-malarial drug approved by the National Malaria Control Programme and was then excluded from the study.

### Treatment complement to improve absorption

To facilitate intestinal absorption of AL, lipid-rich food in the form of sweetened milk concentrate was given to young children (six to 36 months) and groundnut paste to older children (37–59 months) [18,19,23,24].

### Clinical follow-up

Patients were required to come to the health clinic on days 0, 1, 2, 3, 7, 14, 21 and 28 for examination and at any time they felt unwell. Venous blood was collected on day 0, and blood from a finger prick was obtained on each day of follow-up to prepare thin and thick smears for microscopy and a filter paper blood spot for DNA analysis. The patients or guardians were asked about drug consumption since the last clinic visit. Individuals for whom treatment failed were treated according to the National Malaria Control Programme guidelines.

### Microscopy

After thin films were fixed with methanol, both thin and thick smears were stained with 4% Giemsa for 20 min. The smears were read at the enrolment site, and all slides were re-read by another technician who was unaware of the results of the previous analysis at the Institut Pasteur de Bangui. In case of a discrepancy, a third reading was done, and the result agreed by at least two technicians was considered for the analysis. A minimum of 100 microscopic fields were examined, and parasite density was calculated by counting the number of asexual parasites per 200 white blood cells and adjusting for the total white blood cell count by the standard approximation method (40 × number of parasites per 200 white blood cells on a thick film). Thin blood smears were also examined to determine the *Plasmodium* species.

### Differentiation of persistence of infection and reinfection

To identify treatment failures correctly, a clear distinction had to be made between reinfection and recrudescence for patients with recurrent parasitaemia after day 7. *P. falciparum* parasites present in blood samples collected during the trial (on day 0 and the day of parasitaemia recurrence) were genotyped by polymerase chain reaction (PCR) for the parasite merozoite surface protein genes *msp1* and *msp2* and the glutamate-rich protein (*glurp*) [25]. The alleles of the parasites present in the two samples were compared to differentiate persistence of infection (i.e. recrudescence when the alleles of each gene were identical) from re-infection (i.e. different alleles in at least one genes). The latter was not considered to represent failure.

### Determination of transaminases

The levels of alanine and aspartate aminotransferase were measured with an ABX Pentra 400 reader, according to standard biochemical protocols, in blood samples collected from patients treated with combination treatments containing AQ (AS-AQ and SP-AQ) obtained on days 0, 1 and 3.

### Study outcomes

The therapeutic outcome was classified on day 28 according to the WHO protocol [21]. The primary end-point was the 28-day cure rate, defined as the proportion of patients with an adequate clinical and PCR-corrected parasitological response. Secondary end-points were early treatment failure, late clinical failure, late parasitological failure, adverse events, anaemia (haemoglobin <10 g/dl) and rates of clearance of fever and parasitaemia and gametocyte carriage. Parasite and fever clearances were assessed on days 1, 2 and 3, while gametocyte carriage was assessed on days 0, 3, 7, 14, 21 and 28. An adverse event was defined as a symptom or an abnormal laboratory value not present on day 0 but occurring during follow-up, or present on day 0 but becoming worse during follow-up. Serious adverse events were defined according to the WHO [26].

### Statistical analysis

To demonstrate significant superiority (unilateral two-group Fisher exact test) of any of the artemisinin-based combinations over the SP-AQ regimen at risk *p* = 0.05, 57 individuals per treatment group would give a power of 99, 90 and 73% if the clinical and parasitological response rate was 80% in the SP-AQ group and 100, 97.5 and 95% in the three ACT groups. Moreover, if the observed clinical and parasitological response rate was 100%, the lower limit of the exact 95% confidence interval (CI) would be 94%. Assuming a 10% non-accessibility rate (e.g. lost to follow-up), we planned to enrol 251 children (about 63 per treatment group). Data from the two study sites were pooled.

The intention-to-treat population analysis comprised all randomized individuals. Children lost to follow-up and who withdrew were considered possible treatment failures. The per-protocol population analysis comprised all children who took the study medication and attended follow-up to the time of treatment failure or the end of the study (28 days). Children lost to follow-up or who withdrew, including those who violated the protocol, were excluded from the per-protocol analysis. The primary analysis was based on the 28-day PCR-corrected efficacy in both intention-to-treat and per-protocol populations. Baseline and safety analyses were done for the intention-to-treat population.

Data were double-entered, validated with Microsoft Access and analysed with STATA version 10.0 (StataCorp College Station, Texas 77845, USA). Chi-squared and Fisher exact tests were used to compare categorical variables. A Kruskal-Wallis rank test was performed to compare quantitative data for the four treatment groups (parasitological clearance and temperature resolution of fever), and the Wilcoxon matched-pairs signed-ranks test was used to test the equality of matched pairs of quantitative observations. P values <0.05 were considered statistically significant.

### Ethical clearance

The protocol was reviewed and approved by the Scientific Committee of the Faculty of Health Sciences of the University of Bangue, CAR and authorized by the Ministry of Health (No. 14/UB/FACSS/CSCVPER/09). For all patients, written informed consent was provided by a parent or guardian.

## Results

### Baseline characteristics

**Figure 1:**
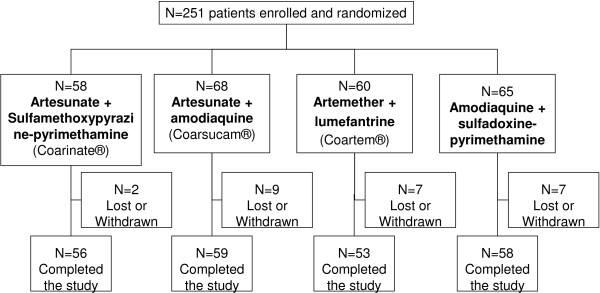
Trial design.

The first patient was enrolled in May 2008, and the study was completed in May 2009. The random assignment to the four treatment groups of the 251 participants for whom slide analysis showed the presence of *P. falciparum* is presented in Figure 1. Of the 251 participants, 226 completed the study (90%); 10% were lost to follow-up, withdrew from the study or were withdrawn from the per-protocol analysis. Demographic, parasitological and clinical characteristics at baseline did not differ significantly between treatment groups (Table 1).

**Table 1:** Baseline characteristics of children at enrolment in a clinical trial of the efficacy of three artemisinin-containing combinations therapies with amodiaquine + sulphadoxine–pyrimathamine (AQ-SP)

	**AS-SMP**	**AS-AQ**	**AL**	**AQ-SP**	***p***
	**N = 58**	**N = 68**	**N = 60**	**N = 65**	
Age (months)					0.396
Median	43	34	40	36	
Interquartile range	27-51	25-49	27-48	23-52	
Minimum, maximum	7-59	7-59	3-58	2-59	
Gender					0.235
Male (n (%))	27 (47%)	39 (57%)	38 (63%)	32 (49%)	
P. falciparum(/μl)					0.853
Median	17 000	18 509	16 471	14 400	
Interquartile range	8200-60 000	6480-9 814	7200-58917	8000-36 000	
Temperature at day 0 (°C)					0.198
Median	38,7	38.9	39.2	39.1	
Interquartile range	38.3-39.7	38.3-39.9	38.4-40.2	38.4-39.7	

*AS-SMP*, Artesunate + sulphamethoxypyrazine-pyrimethamine (Coarinate®); *AS-AQ*, Artesunate + amodiaquine (Coarsucam®); *AL*, Artemether + lumefantrine (Coartem®); *AQ + SP*, Amodiaquine + sulphadoxine-pyrimethamine; *N*, Effective number.

### Efficacy

The day-28 PCR-corrected cure rates and their 95% CIs in the per-protocol analysis ranged from 92% with AL to 100% with SP-AQ (Table 2), with no statistically significant difference among the four treatments (*p* = 0.133). As expected, the 28-day cure rates in the intention-to-treat and in the PCR-uncorrected per-protocol analyses were lower than those in the PCR-corrected per-protocol analysis. On day 28, the PCR-corrected cure rates and their 95% CIs in the intention-to-treat analysis were: 80% (68–89%), 86% (75–94%), 81% (70–89%) and 88% (77–95%) for the AL, AS-SMP, AS-AQ and SP-AQ groups, respectively, with no significant difference among the groups (*p* = 0.578). The day-28 PCR-uncorrected cure rates in the per-protocol analysis also showed no significant difference in efficacy (*p* = 0.077): 91% (79–97%), 86% (74–94%), 93% (84–98%) and 98% (91–100%) for the AL, AS-SMP, AS-AQ and SP-AQ groups, respectively.

**Table 2:** Efficacy of three artemisinin-containing combinations therapy in comparison with amodiaquine + sulphadoxine–pyrimethamine (AQ-SP) on day 28 after PCR correction, per-protocol analysis

		**AS-SMP N = 54**	**AS-AQ N = 59**	**AL N = 52**	**AQ-SP N = 57**	**p-value**
Per protocol analysis (with PCR correction)						
	ETF					
	n % (95% CI)	1 2% (0–10)	0 0% (0–6)	0 0% (0–7)	0 0% (0–6)	
	LCF					
	n % (95% CI)	2 4% (0–13)	3 5% (1–14)	2 4% (0–13)	0 0% (0–6)	
	LPF					
	n % (95% CI)	1 2% (0–10)	1 2% (0–9)	2 4% (0–13)	0 0% (0–6)	
	ACPR					0.133
	n % (95% CI)	50 93% (82–98)	55 93% (84–98)	52 92% (81–98)	57 100% (94–100)	
Intention to treat analysis (without PCR correction)						
	ETF					
	n % (95% CI)	1 2% (0–9)	0 0% (0–5)	0 0% (0–6)	0 0% (0–6)	
	LCF					
	n % (95% CI)	6 10% (4–21)	3 4% (1–12)	2 3%(0–12)	0 0% (0–6)	
	LPF					
	n % (95% CI)	1 2% (0–9)	1 1% (0–8)	3 5% (1–14)	1 2% (0–8)	
	ACPR					
	n % (95% CI)	48 83% (71–91)	55 81% (70–89)	48 80% (68–89)	57 88%(77–95)	0.058

*AS-SMP*, Artesunate + sulphamethoxypyrazine-pyrimethamine (Coarinate®); *AS-AQ*, Artesunate + amodiaquine (Coarsucam®); *AL*, Artemether + lumefantrine (Coartem®); *AQ + SP*, Amodiaquine + sulphadoxine-pyrimethamine; *N*, Effective number; *CI*, Confidence interval.

**Figure 2:**
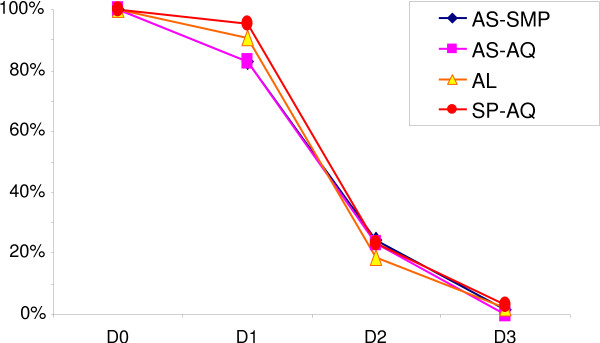
**Characteristics of patients during the first three days after the start of treatment: proportion of patients with parasites, *p *** **< 0.05.**

Parasite clearance rates in each treatment group are presented in Figure 2. The prevalence of infection on day 3 after treatment was 2% (1/53), 2% (1/56), 0% (0/59), and 2% (1/58) in the AL, AS-SMP, AS-AQ and SP-AQ groups, respectively; there was no statistically significant difference among the groups on days 1, 2 and 3.

**Figure 3:**
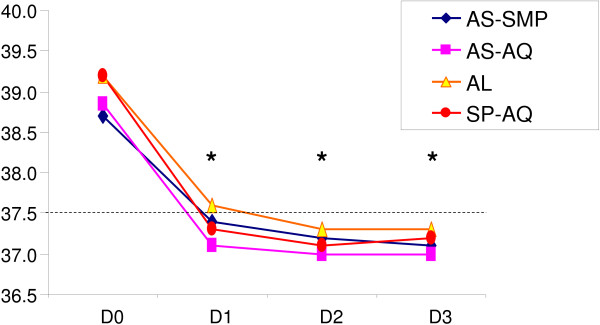
**Characteristics of patients during the first three days after the start of treatment: median body temperature, *p *** **< 0.05.**

Resolution of fever was seen within three days in all treatment groups, although the axillary temperature was always lower after AS-AQ and higher after AL (Figure 3). The difference in fever clearance with the four treatments was significant, with the lowest axillary temperature after AS-AQ and the highest after AL on all three days. Indeed, the mean body temperatures after AL, AS-SMP, AS-AQ and SP-AQ were 37.6°C (37.4-37.8), 37.4°C (37.2-37.6), 37.2°C (37.0-37.4) and 37.3°C (37.1-37.5) on day 1 (*p* = 0.017); 37.3°C (37.2-37.4), 37.2°C (37.1-37.3), 37.0°C (36.9-37.1) and 37.1°C (36.9-37.2) on day 2 (*p* = 0.022); and 37.3°C (37.2-37.5), 37.1°C (37.0-37.3), 37.0°C (36.9-37.1) and 37.5°C (37.0-37.7) on day 3 (*p* = 0.035).

### Tolerability and safety

No deaths and no cases of severe malaria were seen during the study. All adverse events observed were mild and consisted mainly of gastrointestinal symptoms (vomiting, diarrhoea, abdominal pain and anorexia), fatigue and pruritus (Table 3).

**Table 3:** Frequency of mild-to-moderate adverse events on days 1–7 (related or not to treatment) with three artemisinin-containing combinations therapies in comparison with amodiaquine + sulphadoxine–pyrimathamine (AQ-SP)

	**AS-SMP**	**AS-AQ**	**AL**	**AQ-SP**	***p***
	**N = 58**	**N = 68**	**N = 60**	**N = 65**	
At least one adverse event	24	35	17	32	0.037
% (95% CI)	41% (29–55)	51% (39–64)	28% (17–41)	49% (37–62)	
Vomiting	10	12	4	12	0.177
Fever	11	14	8	6	0.143
**Fatigue**	**4**	**9**	**3**	**16**	**0.006**
Abdominal pain	3	8	2	9	0.112
Diarrhoea	4	6	4	5	0.976
Anorexia	3	4	3	8	0.377
Pruritus	2	4	0	4	0.222
Drowsiness	0	0	0	2	0.175
Aphthous stomatitis	0	1	0	1	1.000
Headache	1	0	1	0	0.359
Other*	6	7	3	6	0.666

*AS-SMP*, Artesunate + sulphamethoxypyrazine-pyrimethamine (Coarinate®); *AS-AQ*, Artesunate + amodiaquine (Coarsucam®); *AL*, Artemether + lumefantrine (Coartem®); *AQ + SP*, Amodiaquine + sulphadoxine-pyrimethamine; *N*, Effective number; *CI*, Confidence interval. In bold: the only adverse event for which statistically significant differences (p <0.01) are observed between the treatments.

*Intestinal, respiratory, skin infections, pain, anaemia, cough, injuries, skin rash.

**Figure 4:**
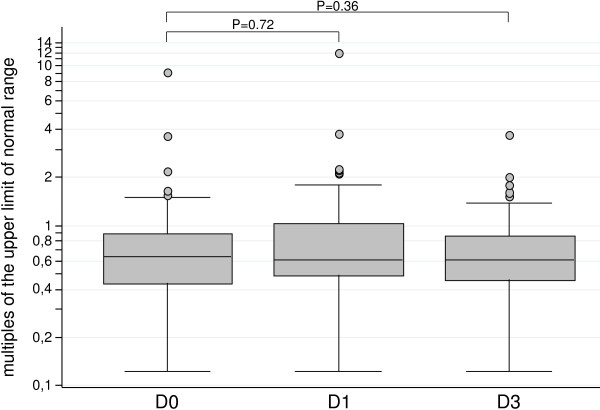
Concentrations of serum liver enzymes, expressed in multiples of the upper limit of the normal range, on days 0, 1 and 3 in 81 patients given amodiaquine treatments: results of alanine aminotransferase (ALT).

**Figure 5:**
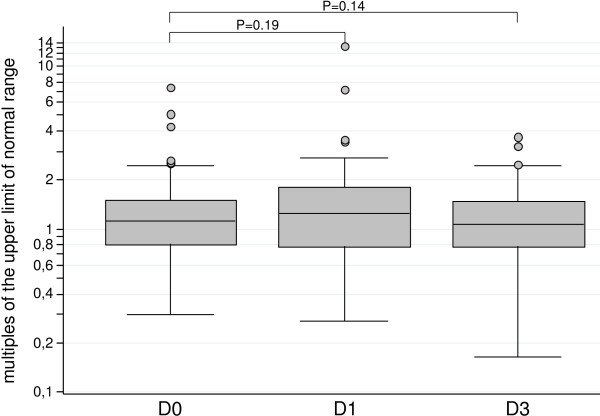
Concentrations of serum liver enzymes, expressed in multiples of the upper limit of the normal range, on days 0, 1 and 3 in 81 patients given amodiaquine treatments: results of aspartate aminotransferase (AST).

Fatigue was significantly more frequent in the two groups of patients receiving AQ (9% with AS-AQ and 16% with SP-AQ; *p* = 0.006). Abdominal pain was also more frequent in AQ users, and vomiting was about three times more frequent in the groups receiving AS-SMP (10%), AS-AQ (12%) and SP-AQ (12%) than in those given AL (3%), but the differences were not statistically significant. In the two groups receiving AQ, values more than three times the normal were found for alanine aminotransferase on day 0 in one child and on day 1 in one child (Figure 4) and for aspartate aminotransferase in six children on day 1 and in three children on day 2 (Figure 5). None of these abnormal biological values was clinically significant. There was no statistically significant increase in aminotransferase activity between day 0 and day 3 (Figures 4 and 5).

## Discussion

This comparative evaluation of four treatments shows satisfactory, comparable efficacy of AL, AS-SMP, AS-AQ and SP-AQ. Only slight differences in cure rates and parasite clearance rates were seen, which were not statistically significant. Significant differences in defervescence were found, AL being the less clinically efficient drug. Thus, this study confirms the efficacy of ACT for the treatment of uncomplicated malaria in children, as reported previously [1,27,28]. As the study was designed to demonstrate the superiority of ACT with respect to SP-AQ, we did not investigate the non-inferiority of any of the drugs.

No clinically significant hepatic toxicity, agranulocytosis [29-32] or serious other side-effects were seen. No significant increase in alanine or aspartate aminotransaminase activity was observed between day 0 and day 3. AQ-containing combinations were associated with more occurrences of fatigue than other drugs.

The finding that treatments containing SP or its derivative SMP (both drug are antifolates) did not increase cure rates significantly in comparison with the other two combinations is puzzling, as resistance to SP in the CAR was found to be high *in vitro*, i.e. up to 22.8% in 2004 [13,33] and would not have diminished subsequently, as SP is still commonly used, despite official recommendations.

Other studies have made similar observations. For example, in Rwanda [34] and the United Republic of Tanzania [35], at least 25% resistance of *P. falciparum* to SP was found, but assessment of the clinical efficacy of combinations containing SP showed adequate clinical and parasitological response rates of 90.3% [36] and 94% [35] in the two countries, respectively. Similarly, AS-SMP (Co-arinate® co-blister) resulted in high adequate clinical and parasitological response rates (96.6%) in the United Republic of Tanzania, Benin and Ghana [37,38]. Despite the high efficacy of AS-SMP and SP-AQ observed in the present study, as in nearby countries, the use of antifolates even in combination with ACT in endemic areas, where the rate of mutations associated with resistance to this family of drugs (in *dhfr* or *dhps*) is high, is not recommended [26]. The prevalence rate of these mutations is yet to be tested in the CAR. It has been also suggested that, in areas with high resistance to pyrimethamine alone or with sulphadoxine, combinations with AS might not be effective [39]. Artemisinin derivatives should not be prescribed alone in order to prevent the selection of resistance; therefore, in a subsequent study molecular biology will be used to determine the prevalence of point mutations in the genes *Pfcrt, Pfmdr, Pfdhfr* and *Pfdhps*, which are associated with resistance to chloroquine, mefloquine, pyrimethamine and sulphadoxine, respectively.

## Conclusions

The efficacy of the three artemisinin-based combinations evaluated did not differ significantly, and they were well tolerated, although AL appeared to have a slight advantage in terms of tolerability and to be associated with slower defervescence. This finding should be confirmed in a larger trial. Given the potential development of resistance to SP and SMP and international recommendations, both AS-AQ and AL could be used in the CAR.

The results of this study should be taken into account by the CAR National Malaria Control Programme in recommending the most appropriate ACT for the country. Further studies should be conducted to evaluate malaria treatment and to reinforce good practice by medical staff.

## Abbreviations

ACT: Artemisinin-based combination therapy; AL: Artemether–lumefantrine; AQ: Amodiaquine; AS: Artesunate; CAR: Central African Republic; CI: Confidence interval; PCR: Polymerase chain reaction; SMP: Sulphamethoxypyrazine-pyrimethamine; SP: Sulphadoxine–pyrimethamine; WHO: World Health Organization.

## Competing interests

The authors declare that they have no competing interests.

## Authors’ contributions

All authors contributed to the design of the study or assisted with data interpretation. DD coordinated the study and supervised the enrolment and follow-up of patients. SPN enrolled patients. DD conducted molecular analysis for determination of recrudescence or reinfection. DD, AM and CR participated in analysing the data and writing the manuscript, and all authors approved the final version.
